# Dual-Mode and Label-Free Detection of Exosomes from Plasma Using an Electrochemical Quartz Crystal Microbalance with Dissipation Monitoring

**DOI:** 10.1021/acs.analchem.1c04282

**Published:** 2022-01-24

**Authors:** Jugal Suthar, Beatriz Prieto-Simon, Gareth R. Williams, Stefan Guldin

**Affiliations:** † UCL School of Pharmacy, University College London, 29-39 Brunswick Square, Bloomsbury, London, WC1N 1AX, United Kingdom; ‡ Department of Chemical Engineering, University College London, Torrington Place, London, WC1E 7JE, United Kingdom; § Department of Electronic Engineering, Universitat Rovira i Virgili, 43007, Tarragona, Spain; ∥ ICREA, Pg. Lluis Companys 23, 08010, Barcelona, Spain

## Abstract

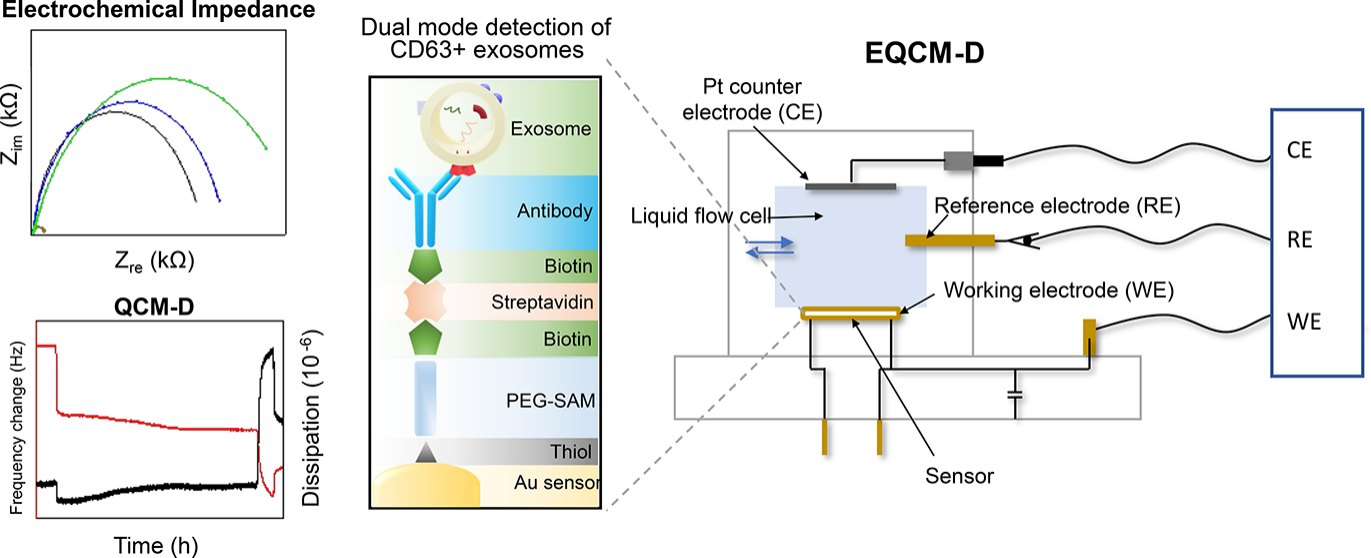

The biomolecular contents of extracellular vesicles, such as exosomes, have been shown to be crucial in intercellular communication and disease propagation. As a result, there has been a recent surge in the exploration of novel biosensing platforms that can sensitively and specifically detect exosomal content such as proteins and nucleic acids, with a view toward application in diagnostic assays. Here, we demonstrate dual-mode and label-free detection of plasma exosomes using an electrochemical quartz crystal microbalance with dissipation monitoring (EQCM-D). The platform adopts a direct immunosensing approach to effectively capture exosomes via their surface protein expression of CD63. By combining QCM-D with a tandem in situ electrochemical impedance spectroscopy measurement, we are able to demonstrate relationships between mass, viscoelasticity and impedance inducing properties of each functional layer and analyte. In addition to lowering the limit of detection (by a factor of 2–4) to 6.71 × 10^7^ exosome-sized particles (ESP) per mL in 25% v/v serum, the synergy between dissipation and impedance response introduces improved sensing specificity by offering further distinction between soft and rigid analytes, thereby promoting EQCM-D as an important technique for exosome analysis.

The search and application of novel biosensing techniques for the detection of exosomal analytes (such as proteins or microRNA) has grown rapidly in recent years. This is fuelled by the discovery that these biomolecules play an essential role in disease progression,^1,2^ but also as potential biomarkers for early disease diagnosis.^3,4^ Minimally invasive liquid biopsies will become a viable prospect if a suitable analytical platform can be coupled with these vesicular sources of rich biomolecular information. In this light, groups have explored a vast array of advanced characterization techniques, including those principled on fluorescence,^5−7^ absorbance,^8−10^ electrochemistry,^11,12^ plasmon resonance,^13,14^ surface enhanced Raman spectroscopy,^15−17^ and, more recently, acoustic resonance in the form of a quartz crystal microbalance with dissipation monitoring (QCM-D).^18−20^

QCM-D is able to successfully distinguish between exosomes and non-exosomal particles based upon their viscoelastic properties as well as their to their mass.^18^ Not only does this offer an additional discriminatory mode of measurement, it exploits immuno-capture principles to specifically detect exosomes based upon expression of the surface protein CD63. Nonetheless, the platform’s limit of detection (LOD) was significantly poorer than competing approaches. One method of improving platform sensitivity is to sense exosomes through an immunosensor transduced via a complementary mode of measurement in tandem with the QCM-D process. An alternative method of detection could also present as a more holistic biosensor, presenting richer insights on fundamental binding phenomena, while providing a point of comparison against the frequency and dissipation outputs from the acoustic approach. It is common to see QCM-D analysis conducted alongside surface plasmon resonance (SPR) spectroscopy, with the rationale being that QCM-D offers cosolvated mass of bound adsorbates while plasmon resonance informs on dry mass, allowing for solvation fraction determination.^21−25^ Recently, a viable route toward dry mass determination by QCM-D has been established via kinematic-viscosity matching.^27^ Another notable synergistic technique is total-internal-reflection-fluorescence microscopy.^26^ Combining QCM-D with in situ ellipsometry measurements supports similar conclusions in addition to greater understanding of the interfacial optical properties.^28−31^

An informative approach, particularly in the field of biosensing, is the combination of electrochemical measurements in conjunction with QCM-D, collectively termed EQCM-D.^32−34^ The technique makes use of a gold coated QCM sensor which also functions as a working electrode (WE) within an electrochemical cell with a conventional three electrode setup. This arrangement enables the conduct of routine electrochemical measurements, such as electrochemical impedance spectroscopy (EIS), cyclic, differential pulse, and square wave voltammetry, while also capturing frequency and dissipation responses simultaneously, in real-time.^35^ The overall result should be an improvement to EIS assays, due to unique information on bound mass, interfacial structural changes, and binding kinetics offered by QCM-D. Similarly, QCM-D is benefited by a greater understanding of how distinct layers alter electron transfer processes at nanomolar sensitivity via EIS.

Traditionally, EQCM-D has been a popular technique for the characterization of electrochemical energy storage and conversion.^36,37^ In a study with clinical relevance, an EQCM cytosensor detected cell surface sialic acid, combining in situ cyclic voltammetry analysis with QCM derived frequency response.^38^ The approach successfully differentiated sialic acid expressed between normal and diabetic patient cells. EQCM-D was showcased as part of a biosensor array for point-of-care detection of dengue fever by targeting the NS1 antigen, with a LOD as low as 10 ng mL^–1^.^39^ Gao et al. employed cyclic voltammetry and acoustic frequency measurements in combination with an immunosensor platform to successfully detect inflammation marker C-reactive protein with a LOD of 0.02 μg mL^–1^. This represented a significant amplification in response compared to an ELISA technique.^40^ Srivastava et al. devised an EQCM platform with an imprinted graphene-starch nanocomposite matrix, using differential pulse voltammetry and frequency for epinephrine detection.^41^ These studies demonstrated the lower LOD that differential pulse voltammetry measurements offer compared to QCM, thereby improving the sensitivity of the platform overall.

In spite of these successful applications, EQCM-D has yet to be explored for the detection of exosomes as a target source of analytes. Recently, however, electrochemical assessments (either on their own or in conjunction with other techniques) have become leading modes of measurement in the exosome field, with many reports exhibiting superior sensitivity toward exosomes than optical based methods of biosensing. Doldan et al. employed amperometry for detecting surface located exosomal proteins using an immunosensor.^42^ Amperometry was also adopted for the iMEX device, which successfully profiled exosomal membrane proteins, including CD63, from cancer patients.^43^ More recently, Moura et al. applied amperometry for cancer-specific exosome markers (CD24, CD44, CD54, CD326).^44^ All three methods use direct or indirect labeling of the exosomal protein, or response amplification through enzymatic digestion. Amperometry was also used in tandem with colorimetric detection, offering a multimodal measurement of placental alkaline phosphatase (PLAP)-positive exosomes.^12^ Square wave voltammetry was implemented as part of an aptasensor platform for exosomal CD63 detection.^45^ Here, a linear potential sweep using a combination of a square wave and staircase potential was applied to an electrode, producing a differential current plot as opposed to more traditional anodic and cathodic peaks. An et al. implemented a differential pulse voltammetry based aptasensor to also detect exosomal CD63 by measuring the reduction in current upon exosome binding.^46^ EIS based detection of CD81-positive exosomes was recently reported, where changes in impedance were found to be proportional to the exosome concentration, as the physical barrier to electron transfer across the WE was increased.^47,48^ EIS was also combined with voltammetry as part of an aptasensor platform to sensitively detect CD63-positive exosomes in human serum.^10^ While these reports did not assess EIS performance in more complex matrices, they lay the foundation for the implementation of EIS as the electrochemical method of choice for the investigation reported herein.

In this work, a method for EQCM-D detection of CD63-positive exosomes is presented. This combines principles of QCM-D and EIS in an attempt to conduct label-free electrochemical transduction of binding events, that can be compared with changes in viscoelasticity and adsorbed mass at the working electrode surface, offering advancements to both modes of assay. Initially, this work determines the most appropriate electrochemical model to fit against the EIS data. Once established, comparisons are made between impedance spectroscopy, frequency and dissipation outputs from the EQCM-D platform. This methodology looks to support measurements for each stage of immunofunctionalisation and immunosensing process (from sensor fabrication to analyte detection). Findings help to understand the influence of molecular rigidity and softness of different layers on EQCM-D response. Furthermore, the sensitivity and specificity of the EIS technique are explored against a range of exosome concentrations in both buffer and complex media.

## Experimental Section

### Materials

Materials were sourced as follows: antimouse detection module for a Western blot WES machine and 12–230 kDa WES separation modules were acquired from Protein Simple (Bio-Techne, Minneapolis, MN). For isolation and sample preparation, qEV original size-exclusion chromatography (SEC) columns (Izon Science, UK), 0.45 μm filters (Merck Millipore, U.S.), HEPES buffered saline (HBS, 0.01 M HEPES, pH 7.4, 0.15 M NaCl) (GE Healthcare Life Sciences, Sweden), Amicon Ultra-15 centrifugal filters (Merck Millipore, U.S.), 100 nm polystyrene beads (Thermofisher Scientific, UK) and RIPA buffer (Sigma-Aldrich, St. Louis, MO) were used. Mouse monoclonal anti-Alix (634 502, Biolegend UK), mouse monoclonal anti-CD63 (353 013, Biolegend UK), mouse monoclonal anti-CD9 (31 202, Biolegend UK), mouse monoclonal biotinylated anti-CD63 (353 017, Biolegend UK) and biotin-IgG isotype control antibody (400 103, Biolegend UK) were acquired for Western blot and immunosensing experiments. Ferrocyanide (>98.5%, Honeywell) and ferricyanide (99%, ACROS Organics) were employed as the redox couple for EIS measurements. Human plasma and streptavidin were purchased from Sigma-Aldrich, St. Louis, MO. 5-MHz gold coated QCM sensors were purchased from QuartzPro, Sweden.

### Exosome Isolation and Characterization

#### Size-Exclusion Chromatography

SEC was implemented as the exosome isolation technique from human plasma. The source plasma was first filtered with a 0.45 μm filter (Merck Millipore, U.S.). Thirty mL of clarified plasma was subsequently concentrated using Amicon Ultra-15 centrifugal filters with a 10 kDa pore size cutoff (Merck Millipore, U.S.). The filters were spun at 4000*g* for 30 min at 4 °C. Postspin, 0.5 mL of concentrated filtrate was loaded onto a qEV 35 nm SEC column (Izon Science, UK). 0.2 μm filtered HEPES buffered saline (HBS, 0.01 M HEPES, pH 7.4, 0.15 M NaCl) (GE Healthcare Life Sciences, Sweden) was used as the eluting buffer at a flow rate of 1 mL min^–1^. Twenty 1 mL fractions were collected and stored at −80 °C.^18^

#### Western Blot Analysis of Final Isolate

Validation of the SEC protocol was conducted by verifying exosome presence through Western blot analysis using capillary gel electrophoresis. Based upon previously reported SEC isolation protocols, SEC fractions 4, 5, 6, 7 were considered for onward protein characterization for exosome presence.^49,50^ Exosomal proteins Alix (97 kDa), tetraspanin CD63 (57 kDa) and CD9 (24 kDa) were probed by chemiluminescent immunoassay, using mouse monoclonal anti-Alix, mouse monoclonal anti-CD63 and mouse monoclonal anti-CD9 as primary antibodies. The Western blot run was conducted as per the manufacturer’s instruction (see Supporting Information (SI) for details).

#### NTA Analysis of SEC Fractions

Based upon the protein identification from the Western blot, the sixth isolation fraction was chosen for concentration and hydrodynamic size characterization of particulates nanoparticle tracking analysis (NTA) with the Nanosight LM10 instrument (Malvern Instruments, UK). The machine was calibrated with 100 nm polystyrene beads (Thermofisher Scientific, UK) prior to fraction assessment. Measurement specifications were as follows: 532 nm green laser, five videos per fraction, 60 s video length, shutter speed of 25–32 ms, camera gain of 400, camera level 15, lower threshold of 910 and higher threshold of 11 180. Captured videos were processed using the NTA software version 3.2, a detection threshold of 5, auto settings for blur, minimum track length, and minimum particle size. Measurements were carried out in static mode at room temperature.^18^

### EQCM-D Measurements

#### General Methods for EQCM-D Apparatus Setup and Sample Preparation

All EIS measurements reported herein were conducted using a Q-Sense Electrochemistry Module from Biolin Scientific (Sweden), in tandem with a Q-Sense Analyzer instrument and a Gamry (UK) Reference 600 Plus potentiostat. The system used gold coated QCM sensors as the WE, a platinum counter electrode (CE) and Ag/AgCl reference electrode (RE) as part of a conventional three-electrode system. Data was acquired with the Gamry Instrument Framework (v7.07) software and analyzed using Gamry Echem Analyst (v7.07) software. When fitting the chosen circuit model to the captured data, parameters were chosen based on well-defined physical processes.

EIS experiments were all carried out with a frequency scan range of 10^–1^ Hz to 10^5^ Hz at a 5 mV AC amplitude. The detection area was set at 0.79 cm^2^. An equimolar solution of 5 mM of K_3_[Fe(CN)_6_]/K_4_[Fe(CN)_6_] in 0.1 M KCl was used for all measurements. Modified Randles cell circuit models served to fit against EIS data and an optimal model was selected. Impedance was determined after the formation of each layer, following the addition of electrolyte into the chamber. The flow of electrolyte through the chamber was paused during measurement acquisition. EIS were captured in tandem with QCM-D response in all instances.

All QCM-D measurements were performed on a Q-Sense E4 instrument (Biolin Scientific, Sweden). Analysis of frequency and dissipation response was conducted using the QTools software, version 3.0.17.560 (Biolin Scientific, Sweden). Changes in resonance frequency (Δ*f*) were recorded from the third, fifth, seventh, ninth and 11th overtones. The presented data relates to the fifth overtone, with variation of Δ*f* between overtones being 10% or less. In all instances, samples were degassed prior to exchange in the QCM flow module and AT-cut, 5-MHz gold coated quartz crystal sensors with a 0.79 cm^2^ active area (Biolin, Sweden) were used.

To ensure reproducibility of each process, all analytes were prepared using the same degassed stock solutions to minimize impact of buffer properties during sample exchange in observed responses. These were prepared to identical volumes (0.25 mL per sensor). All reagents were sourced from the same suppliers throughout the study to avoid influences of differing characteristics or quality. In all cases, the analyte was flowed at 10 μL min^–1^ and a sensor was reserved to monitor for drift induced by buffer exchange. Drift in frequency and dissipation exceeding 1.5 Hz/h and 2 × 10^–7^/h, respectively, was to be deemed excessive; however, this was not ever encountered. All results presented are corrected for background noise as determined by responses on an isotype control functionalized sensor. A HBS buffer rinse was performed after the addition of every functional layer or analyte, with frequency and dissipation responses reported net or post-HBS rinse, to remove the response bias of weakly bound analytes and any solvent specific effects.^18^ For the purposes of this study, baseline is defined as the response achieved immediately prior to the addition of an analyte.

#### Sensor Functionalization

An affinity based immunosensing approach was employed as reported by Suthar et al.^18^ A 1 mM ethanolic solution of SH-PEG (2 kDa)-Biotin and spacer molecule SH-OEG (800 Da)-COOH at a 1:9 mol/mol ratio was flowed across the sensor surface at 7.5 μL min^–1^ overnight to form a self-assembled monolayer (SAM). Subsequently, a 100 μg mL^–1^ solution of streptavidin (SAv) was flowed across the sensor surface at 10 μL min^–1^, followed by a rinse step of HBS buffer at 80 μL min^–1^. Twenty μg mL^–1^ of mouse monoclonal biotinylated anti-CD63 was immobilized on the surface at 10 μL min^–1^, followed by another rinse step and response stabilization for 30 min prior to sample addition.

#### EQCM-D Detection of CD63-Positive Exosomes

The QCM-D immunosensor performance toward spiked CD63-positive exosomes was assessed in tandem with electrochemical measurement (EQCM-D). Sensitivity toward CD63-positive exosomes in HBS buffer and 25% v/v serum was tested using titrated concentrations of ESPs verified by NTA. The following concentrations were assessed: 5 × 10^7^, 7.5 × 10^7^, 1 × 10^8^, 2.5 × 10^8^, 5 × 10^8^, 7.5 × 10^8^, 1 × 10^9^, and 2.5 × 10^9^ ESPs mL^–1^. To complete our sensitivity and specificity assessments, these samples were applied to control sensors that employed a biotin-IgG isotype control antibody in place of biotin-anti-CD63. This helped to determine the signal-to-noise ratio (SNR), statistical significance, solvent effects and noise in our setup.

In this work, LOD is defined as the concentration eliciting a SNR of 3 as per recommendation by Shrivastava et al., with SNR being calculated as a response ratio of the target sensor and control sensor.^51^ For the purposes of this study, dynamic range is defined as the range of concentrations between the limit of detection (LOD) and the concentration where the saturation commences (nonlinearity appears).

## Results and discussion

**Figure 1:**
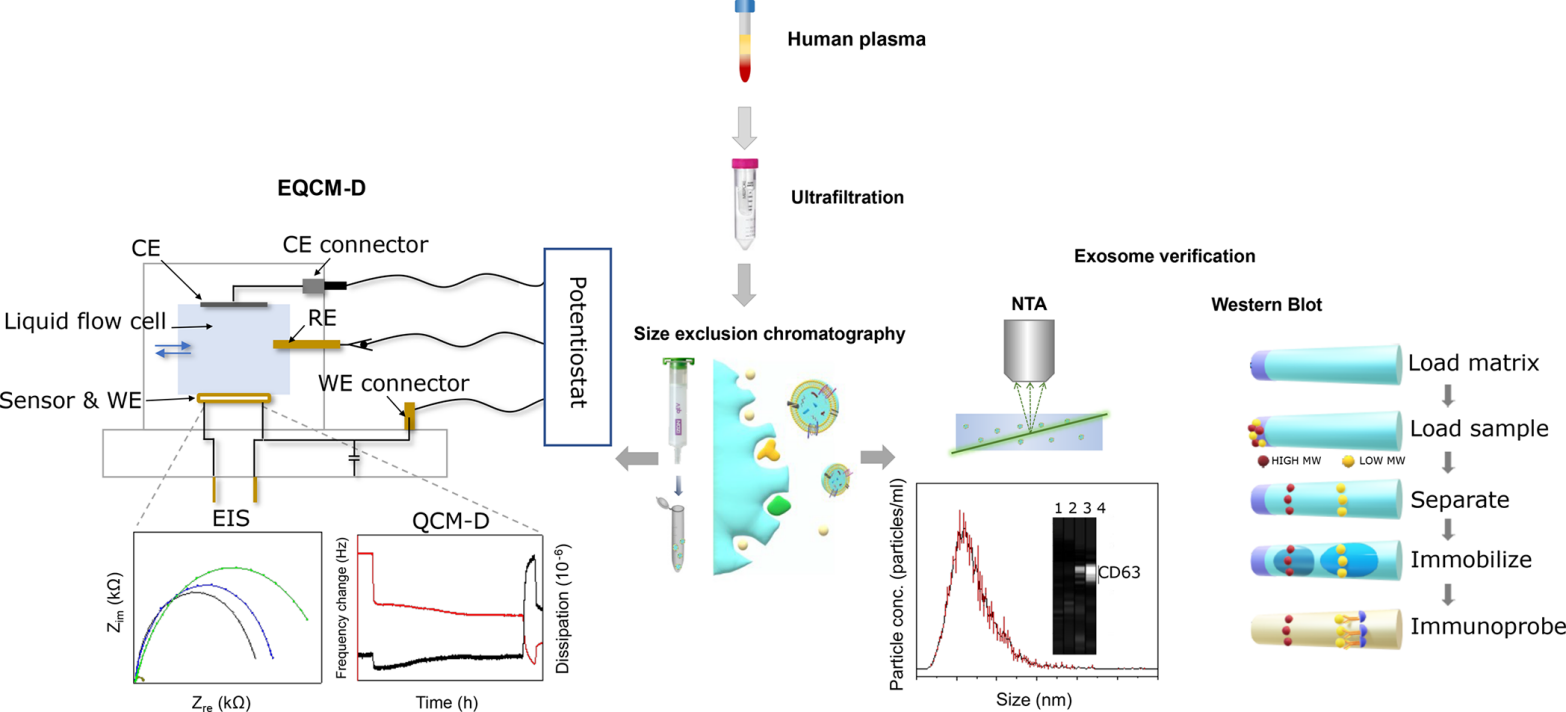
Overview of the adopted experimental approach. CD63-positive exosomes were isolated from human plasma using SEC, prior to exosome verification by nanoparticle tracking analysis, Western blot and subsequent biosensing via the EQCM-D platform with the instrumental setup shown. CE: counter electrode, RE: reference electrode, WE: working electrode, EIS: electrochemical impedance spectroscopy, EQCM-D: electrochemical QCM-D, NTA: nanoparticle tracking analysis.

Advancing the detection of plasma derived exosomes is imperative to enable their full exploitation in diagnostic settings. We looked to expand the exosome analytical toolkit by employing a dual-mode method of analysis that combines in situ EIS measurements with QCM-D. The result is an immunosensor which was transduced through both bulk acoustic wave and electrochemical principles. A summary of the experimental work flow is displayed in Figure 1.

### SEC Isolation of Plasma Exosomes

Exosome enriched proteins such as cytosolic Alix, transmembrane CD63 and CD9 were probed using Western blot in a capillary format (SI Figure S1A). Alix and CD9 were seen to be prominently expressed in particles present in fractions 5 and 6, with smaller concentrations of CD9 also seen in fractions 4 and 7. CD63 was solely detected in fractions 6 and 7. As all three exosomal markers were present in fraction 6, this isolate was selected for downstream characterization and biosensing due to favorable protein expression. These results underlined the heterogeneity of exosome composition, which is further influenced by the source media, where differing parent cells incite variable marker expression in relation to their size.^52−54^ NTA analysis of fraction 6 found over 81% of particles falling within the exosomal size range, with a mean size of 127 nm (SI Figure S1B). Collectively, this data confirms that plasma exosomes expressing the target CD63 protein were successfully isolated with the applied SEC protocol.^55−57^ It also ensures the analyte possesses the molecular composition required for the EQCM-D immunosensing approach, which was based upon surface capture of CD63.

### EIS Characterization of Immunosensor Fabrication

To reliably determine the resistance to charge transfer (*R*_ct_) from the EIS data, an appropriate circuit model was required. SI Figure S2A displays the two different circuit models that were evaluated. The bounded Warburg model and constant phase element (CPE) with diffusion model both account for key components of the electrochemical system. First, as a double-layer capacitance is created at the interface between the conductive gold WE and the adjacent liquid electrolyte, a CPE component was introduced to account for the imperfect capacitor that is the dielectric layer.^58^ Second, the impedance of electron transfer at low frequencies is increased by the long distance of electron diffusion through the bulk phase. This is especially relevant to the system in question due to the large size of bound ESPs, thus it is addressed by the inclusion of a Warburg element. The key difference between the two models is that the bounded Warburg model assumes two time constants, compared to the single time constant of the CPE with diffusion model. The incorporation of two time constants assumes a greater complexity in the dielectric features at the WE surface; that is, it accounts for the varying degree of capacitance that is potentially introduced by multiple sensing layers or layers with high heterogeneity in thickness and coverage.

**Figure 2:**
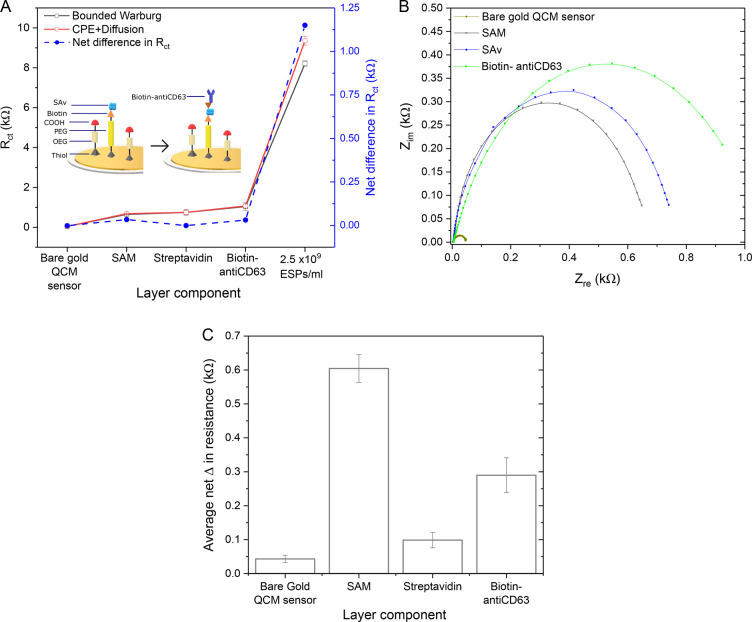
EIS characterization of immunosensor fabrication. (A) Comparing net change in *R*_ct_ for each layer attained from both cell models. Schematic of sensor functionalization approach shown (not to scale). (B) EIS Nyquist plots for each sensing layer fitted with the bounded Warburg model. (C) Bar graph showing mean net change in *R*_ct_ for each layer from (B). Standard deviation determined from three independent experiments.

EIS data captured after the formation of each detection layer were fitted with both models (SI Figure S2B–E). The goodness of fits displayed in SI Table S2 were found to be largely similar between the models for these sensing layers, considering an alpha-level of 1%. However, some advantage was seen with the bounded Warburg model when applied to impedance spectra acquired post-SAv addition (SI Figure S2E), as indicated by the inferior goodness of fit value. Overall, this is expected, as layers comprised of homogeneous small molecules (<15 nm) and that are known to incur small changes in dissipation, such as the mixed-SAM, SAv and antibody, do not induce significant changes in capacitance or increases in diffusion related impedance, thus presenting similar impedance changes between models (Figure 2). Displayed net differences refer to the determined *R*_ct_ of the two models.

Figure 2B illustrates how impedance incrementally increased upon the addition of each layer, demonstrated by the increasing diameter of the Nyquist plot semicircle. As the biomolecular layering at the WE surface changes during the sensing process, this increased the barrier and therefore resistance to charge transfer across WE for current generation. This change in charge transfer relates to the raised impedance of the overall system. Similar impedance increases upon immunosensor fabrication have been reported in literature.^59−61^ It is interesting to note the extent to which *R*_ct_ increased for each sensing layer (Figure 2C). Here, the net changes in resistance is the increase in *R*_ct_ upon analyte addition from the previous (pre-analyte) baseline. While the bare gold sensor displays minimal charge resistance, the mixed-SAM increased the impedance of the system by ∼0.6 kOhm, followed by the anti-CD63 antibody (∼0.3 kOhm) and SAv (∼0.1 kOhm).

A possible explanation for the high impedance induced by the PEG-SAM is that it produced a homogeneous coverage across the entire WE surface, while also assembling with high order and packing density, thereby forming a complete barrier to electron transfer.^62,63^ Furthermore, PEG at the WE surface has been shown to increase the dielectric capacitance, likely contributing to the large increase in impedance.^64,65^ Another consideration is that the mixed-SAM was formed directly on the WE gold surface. Therefore, another reason for the large increase in *R*_ct_ is that the impedance of a double-layer capacitor was more sensitive to electrical potential when changes occur nearer to the WE surface, as it disrupts the ion dense region but also reduces ion permeability. Despite the SAv and antibody layer increasing impedance, they do so to a lesser extent than has been in the case in other studies.^66,67^ This could be a result of layers which are not homogeneously arranged and instead present as a layer of discrete particles, with a suboptimal surface coverage or an incomplete layer. In consequence, certain regions may provide easier access to the underlying SAM for passing ions, lowering the mean *R*_ct_, a notion also advocated by Pali et al.^68^

Crucially, however, a divergence of ∼1 kOhm was seen between the CPE with diffusion model compared to the bounded Warburg model upon application of an ESP solution to the WE surface (SI Figures S2F and Figure 2A), resulting in a significant difference in goodness of fit (SI Table S2). The introduction of heterogeneous particles to the surface, up to 150 nm in size and filled with cytosolic fluid, is likely to have disrupted the dielectric capacitance at the WE, increased the diffusion length and raise the barrier to electron transfer across the WE (also known as tunnelling distance), thereby exaggerating the impedance (in excess of 8 kOhm) at low frequencies compared to the functional layers formed prior (Figure 2A). The overestimation by the CPE with diffusion model could be attributed to the model not sufficiently considering the complexity of capacitance at the surface caused by highly heterogeneous exosome layer, both in terms of coverage and individual size, thus the bounded Warburg model was selected as the optimal approach moving forward for *R*_ct_ determination. EIS data displayed herein shall showcase *R*_ct_1 as the more sensitive parameter.

### Tandem EQCM-D Detection of CD63-Positive Exosomes

**Figure 3:**
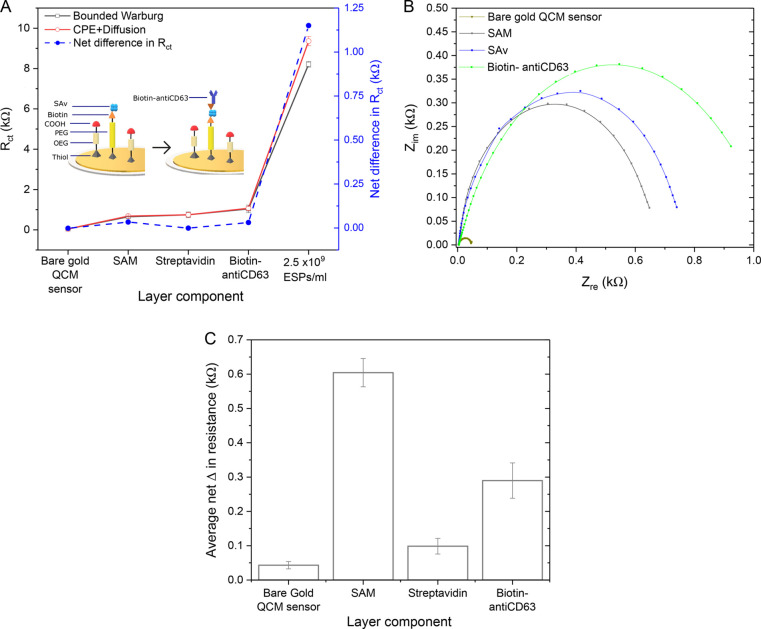
Example of tandem EIS and QCM-D information acquisition. (A) EIS response across entire sensing process (fabrication to ESP detection) captured simultaneously alongside, (B) QCM-D response profile on the same WE for SAM formation (from ethanolic solution) and the addition of (C) SAv, biotin-anti CD63 and 7.5 × 10^8^ ESPS mL^–1^ in HBS buffer. Vertical dashed lines represent the start of respective sample uptake by the peristaltic pump.

The EIS characterization of the sensor fabrication and exosome detection process was acquired in tandem with QCM-D monitoring within a single instrumental setup. Example EIS and QCM-D profiles captured on the same WE are displayed in Figure 3. It is apparent that an increase in impedance coincides with a decrease in frequency upon analyte addition to the sensor surface. The addition of sensor mass upon adsorbate binding reduced the oscillatory frequency of the sensor, while also providing a barrier to electron transfer. Capture of exosomes to the WE resulted in a significant increase in dissipation, due to the raised friction upon oscillation in the adlayer. Moreover, the energy storage and loss of the viscoelastic ESPs amplified the oscillatory decay, in line with results previously reported.^18^ This viscoelastic layer also resulted in the largest increase in system impedance (∼5.5 kOhm). While the large ESP mass was likely a prominent contributor to the EIS response, the size of the particles and their dissipation display a stronger complementarity with the *R*_ct_.

**Figure 4:**
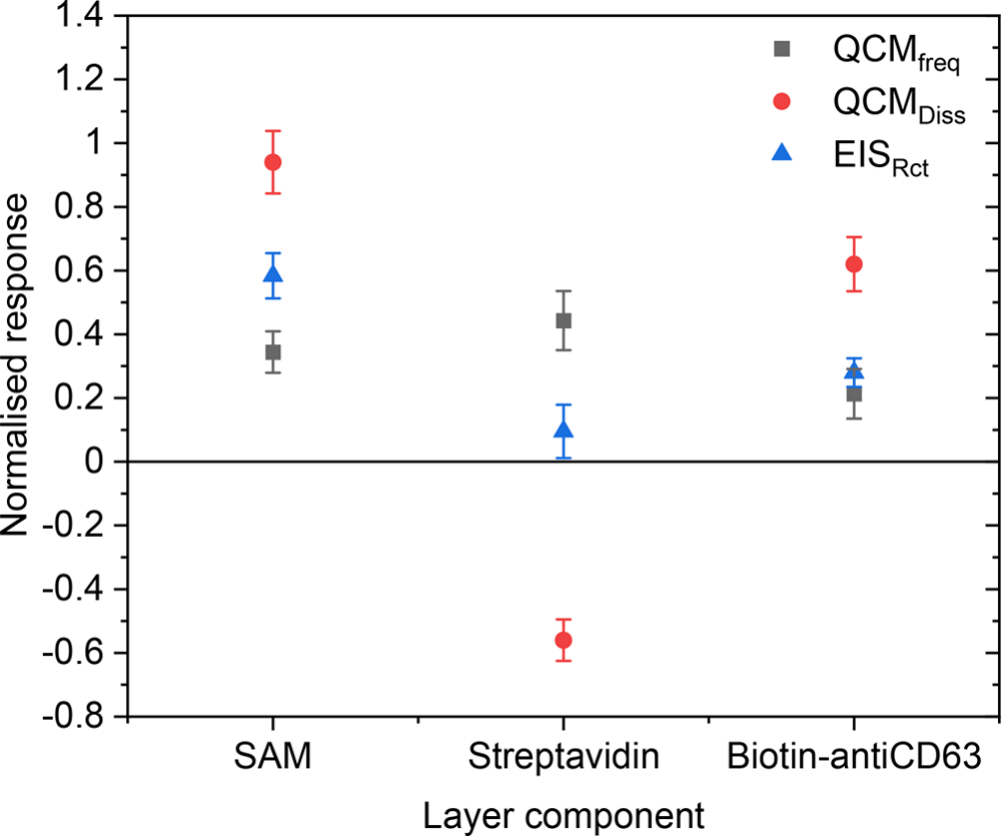
Normalized response comparison upon sensing layer addition between EIS, frequency and dissipation modes of analysis. Responses are taken as a ratio of change from the baseline prior to analyte addition. Standard deviation is determined from three independent experiments.

Response comparison during immunosensor fabrication between the three modes of measurement (EIS, frequency and dissipation) is displayed as a proportional change in Figure 4. Responses were normalized to a baseline before the addition of analyte. This is common practice in QCM-D and EIS analysis to account for intersensor variability. Furthermore, it supports direct comparison in responsiveness of the techniques. It is clear that of the three modes of measurement, QCM-D derived dissipation exhibited superior magnitude of response across all three sensing layers, with significant increases for dissipative layers (SAMs and antibodies) and decreases for non-dissipative layers (SAv). Layers that increased dissipation also seemed to elicit higher proportional responses in impedance rather than frequency. Conversely, SAv which was found to decrease dissipation during oscillatory decay, induced a relatively smaller impedance increase and far lower than QCM derived frequency. These findings further support the proposal that dissipative layers elicit higher impedance at the working electrode surface.

### Sensitivity, LOD and Specificity of EQCM-D Toward CD63-Positive Exosomes

**Figure 5:**
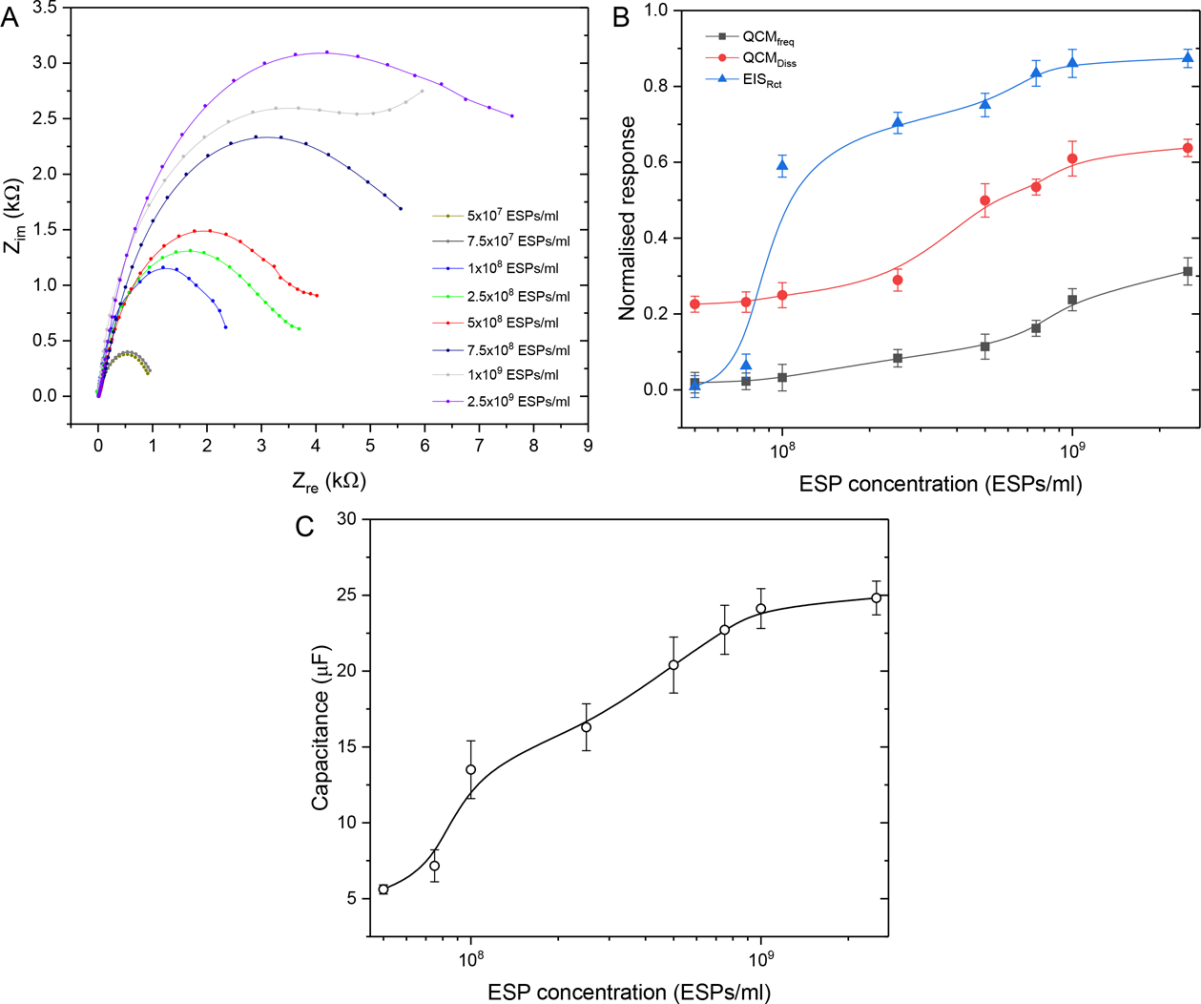
EQCM-D performance against varying concentrations of ESPs spiked in 25% v/v serum. (A) Nyquist plots representing EIS response. (B) Normalized response changes across EIS, frequency and dissipation. (C) Change in system capacitance as exosome concentration increases.

After characterizing EQCM-D responses for the immunosensor fabrication process, the impact of titrated concentrations of ESPs in HBS buffer and 25% v/v serum was explored (SI Figure S3 and Figure 5, respectively). SI Figure S3A and Figure 5A present the corresponding Nyquist plots that show increasing half-circle diameters as ESP concentration increases. The increase in *R*_ct_ is expected, as higher concentrations of ESPs resulted in a greater coverage of the WE surface with particles, which are large and dissipative in nature, raising the tunnelling distance and also the impedance to electron flow for eventual polarization. This was similarly reported by Kilic et al. where increasing concentrations of EVs resulted in *R*_ct_ increments.^47^ A second phase in the form of an upward inflection was seen at lower frequencies for a few of the impedance profiles. This was the effect of Warburg or diffusion related impedance induced by the formation of an exosome layer at the surface, that forced reactants to diffuse greater distances to the electrode surface.^69^ This phenomenon was not apparent across all concentrations, suggesting some heterogeneity of exosome layer coverage between sensors.

Response to these concentrations were compared between EIS, frequency and dissipation via normalized changes from the baseline prior to ESP addition (SI Figures S3B and Figure 5B). A steady increase in response is seen for all measurements along with ESP concentration. At lower ESP concentrations, EIS was seen to be the most responsive of the three techniques. Upon increasing the ESP content, dissipation became more responsive than frequency prior to plateauing along with EIS at the highest concentrations as the detection reached saturation. Once again, we see a correlation between the highly dissipative ESP analyte and a pronounced response in electrochemical impedance, in contrast to a comparatively small frequency change. Furthermore, EIS was seen to be more responsive than dissipation for the highly viscoelastic ESPs, compared to the smaller antibody and SAM adsorbates in Figure 4, where dissipation exhibited the stronger relative responses. It can be argued that the increased electron diffusion path length of the larger sized ESPs has a greater influence on impedance than their acoustic dissipation, compared to the SAM and antibody molecules.

Nonetheless, frequency response was seen to increase slightly along with ESP concentration, which could be accounted for by the fact that frequency is a measure of the cosolvated mass. The solvent coupling includes the liquid in the interstitial spaces between adsorbed ESPs.^70^ The amount of liquid that is trapped is influenced by a few factors. Generally, if the higher coverage of ESPs was creating a homogeneous layer at the sensor surface, it would result in a displacement of entrapped liquid, thus reducing solvent contribution to frequency response.^71^ However, it is likely that even at high ESP concentrations, the surface coverage is incomplete, making it important to consider the hydrodynamically trapped liquid coats surrounding each immobilized particle. It is also known that higher liquid content of the particle results in higher solvent entrapment within the particle layer, which is clearly applicable to the cytosol filled exosomes.^72^ Furthermore, solvent association is seen to be higher for layers comprised of particles with heterogeneous height-to-width ratios. Exosomes already possess variable sizes and are assumed to deform at the WE surface giving low height-to-width ratios, thereby supporting the feasibility of this theory.^13^ Lastly, entrapment is shown to be prevalent where particles have a disparate distribution/lateral organization.^71^ The combination of liquid filled particles, particle deformation and heterogeneity in distribution may all contribute to stronger hydrodynamic effects at higher ESP concentrations, leading to the more pronounced frequency response. The increased trapping of ions within this layer may also be the underlying reason behind the increase in system dielectric capacitance as exosome concentration increases (Figure 5C). Alternatively, the relative plateauing in dissipation response at the highest concentrations could be due to a crowding effect of ESPs at the sensor surface, which reduces the hydrodynamic interaction between particles and inhibits the rocking and translational movements of the bound ESPs where dissipation normally occurs.^72^

Moreover, no significant matrix effects were observed when comparing the performance of the EQCM-D platform to titrated ESP concentrations between HBS buffer and more complex media (25% v/v serum), with the differences in linear response gradient of <10%. This is despite the likely presence of nonspecific particles (proteins, lipoproteins and lipids) that could alter the detection and affect the relative response between the techniques. Figure 5B shows that the introduction of serum into the buffer caused an overall increase in the system dissipation compared to buffer alone (SI Figure S3B), likely due to a small degree of nonspecific binding and the sensitivity to an increase in running buffer viscosity. Crucially, however, the relative outputs from both EIS and QCM-D remain unaffected by non-exosomal artifacts. As the overall response relationships between techniques are unchanged, it points to a high platform specificity, likely attributed to the nonfouling properties offered by the mixed-PEG SAM.^18^

**Figure 6:**
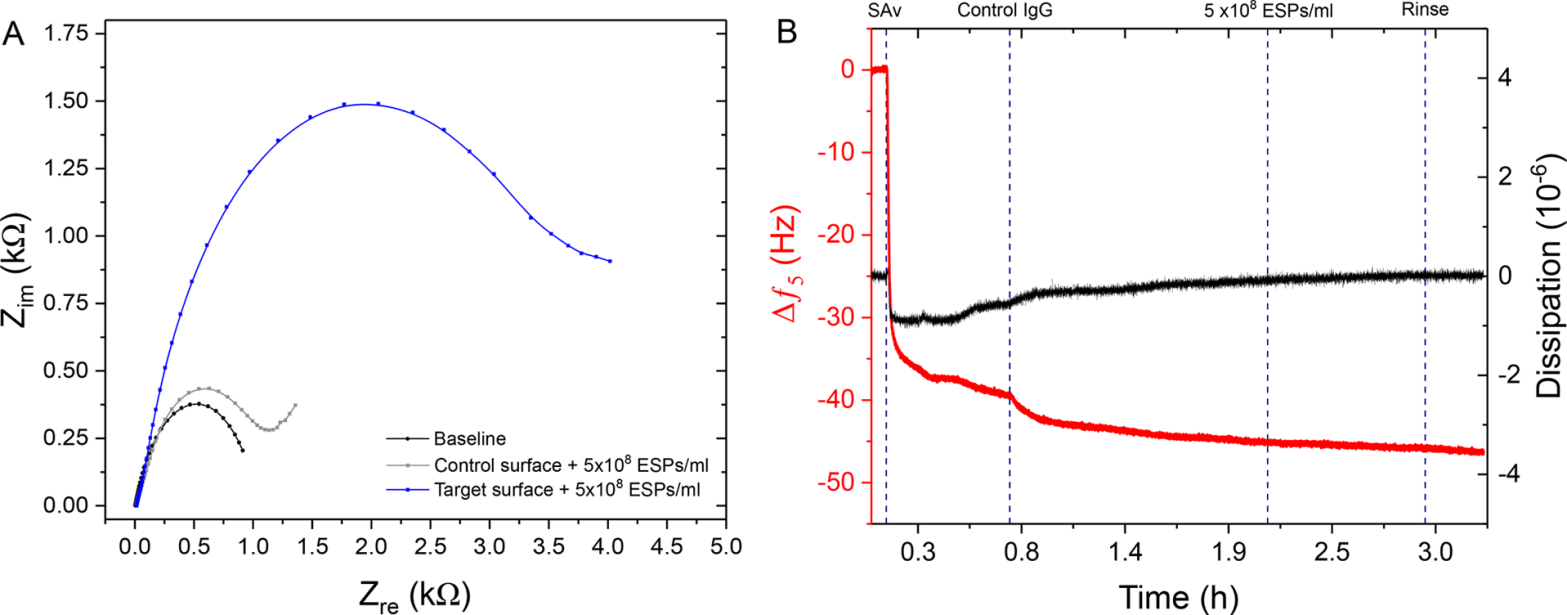
Specificity assessment of the EQCM-D platform. (A) EIS response to 1 × 10^8^ ESPs mL^–1^ on a control surface compared to a target surface. (B) Tandem QCM-D frequency and dissipation response toward 1 × 10^8^ ESPs mL^–1^ captured on a control functionalized sensor.

This notion was further enforced when comparing the target platform response to an ESP sample with a control surface. The control surface had anti-CD63 antibodies replaced with nonspecific IgG antibodies. Figure 6A confirmed a small difference in impedance response between the control surface (1105 Ohms) and a baseline reading (858 Ohms) conducted in just running buffer. The increase in impedance could be attributed to the binding of some nonspecific proteins to the WE surface from the 25% v/v serum matrix. Crucially, an identical sample (1 × 10^8^ ESPs mL^–1^) elicited an impedance reading of 4512 Ohms on the target surface, equating to a SNR of 4.1. This phenomena is also reflected in the tandem QCM-D results in Figure 6B. Minimal increases in dissipation, or decrease in frequency, was seen upon addition of ESPs, demonstrating a low propensity for nonspecific binding.

A full set of isotype control data for the entire range of ESP concentrations in both HBS buffer and 25% v/v serum is displayed in SI Figure S4. The small degree of nonspecific binding introduced by the complex serum is seen across all EPS concentrations and across all modes of measurements. The responsiveness to this nonspecific binding is exacerbated at ESP concentrations >1 × 10^9^ ESPs mL^–1^ for frequency measurement and EIS read-out in particular. This could indicate that the artifactual binding on the control sensor has minimal impact on layer viscoelasticity, hence marginally impacting dissipation response compared to HBS buffer alone. This information supported the generation of SNR across all ESP concentrations (SI Figure S5) in both HBS buffer and 25% serum. Again, it is reasonable to suggest that frequency is most significantly affected by the introduction of more complex media, resulting in a significant reduction in SNR compared to EIS and frequency. While dissipation seemingly displays lower SNR overall, it is the least affected by the increase in matrix complexity. A plateauing of SNR at the highest ESP concentration could indicate signal saturation relative to noise.

The working concentrations of the three analytical principles employed by the EQCM-D platform toward CD63-positive exosomes in 25% buffer were derived from normalized response curves, with the LOD displayed in Table 1. The statistical significance of the target sensor response compared to the control sensor at the respective LOD concentrations is displayed in SI Figure S6. Although frequency response was seen to possess a larger dynamic range of measurement; dissipation and EIS exhibited a superior LOD. Furthermore, as EIS was more effective at minimizing background signal, it delivered a favorable SNR across the range of concentrations, resulting in a lower LOD than dissipation response by approximately half an order of magnitude (Table 1). This aligns with other reports of the more sensitive EIS lowering the LOD of the overall detection system.^39,41,73^ These detection limits are 1–2 orders of magnitude better than some recent biosensing attempts of exosomes, all while being conducted in complex media.^74−78^

**Table 1:** EQCM-D LOD Values.

	LOD (ESPs mL^–1^)		
Sample media	QCM_freq_	QCM_diss_	EIS
HBS buffer	1.71 × 10^8^	1.08 × 10^8^	5.34 × 10^7^
25% v/v serum	2.15 × 10^8^	1.25 × 10^8^	6.71 × 10^7^

Overall, these results underline the capability of the EQCM-D based immunosensor to effectively discern CD63-positive exosomes in a sensitive and specific manner. The clinical significance of these findings includes being able to selectively detect CD63-positive exosomes in minimal sample volumes and biological fluids where average exosome concentrations are lower than native levels.

## Conclusion

In conclusion, we report a significant extension to a previously established QCM-D immunosensing method for CD63-positive exosomes, by incorporating an additional mode of in situ measurement in the form of EIS. Using a circuit model which incorporates two time constants to account for dielectric complexity, we successfully showcase a multimodal approach that offers label-free analysis with high specificity from complex matrices. Uniquely, all this was achieved while using the same sensing surface to detect exosomes based upon their mass, viscoelasticity, and impedance inducing properties. Such an approach allowed for analytical comparison between techniques, which suggested a synergy between EIS and QCM-D, as determined by the magnitude of dissipation by an analyte and resulting increase in impedance at the working electrode. This allowed further discrimination between rigid and viscoelastic particles, which is of particular utility for fluid filled exosome analytes. Besides the complementarity between the techniques, impact was delivered through a lowering of the LOD to 6.71 × 10^7^ ESPs mL^–1^ in 25% v/v serum, compared to 2.15 × 10^8^ and 1.25 × 10^8^ ESPs mL^–1^ for QCM-D frequency and dissipation techniques, respectively.

Ultimately, the strengths of both QCM-D and EIS are enhanced when applied in combination due to the unique insight offered by each technique. This work exhibits a more sophisticated biosensor, with QCM-D offering information on binding kinetics, mass, and interfacial structuring during layer formation, while EIS improves the sensitivity and detection limit of the platform overall. This methodology can now be used to detect a library of exosomal markers with diagnostic potential or for quality control. The authors encourage the research community to incorporate EIS data into the ongoing efforts to derive quantitative exosome information using acoustic-based detection platforms.

## Supporting Information Available

Western blot methodology; Characterization of SEC fraction isolates; Equivalent circuit model evaluation; Goodness of fit model comparison; EQCM-D performance against varying concentrations of ESPs spiked in HBS buffer on a target sensor; EQCM-D response against varying concentrations of ESPs spiked in either HBS buffer and 25% serum using an isotype control sensor; Measurement mode comparison of signal-to-noise ratio (SNR) across titrated ESP concentrations; EQCM-D response comparison between control and target functionalized sensors at each measurement mode’s respective LOD concentration in HBS and 25% serum (PDF)
