# Correction to “Dual-Mode and Label-Free Detection of Exosomes from Plasma Using an Electrochemical Quartz Crystal Microbalance with Dissipation Monitoring”

**DOI:** 10.1021/acs.analchem.2c03078

**Published:** 2022-08-02

**Authors:** Jugal Suthar, Beatriz Prieto-Simon, Gareth R. Williams, Stefan Guldin

We would like to update the content of Figure 3. In the original article, both Figure 2 and Figure 3 are identical. This error was introduced during the production process and after proof correction. The correct version of Figure 3 is shown below.

**Figure 3:**
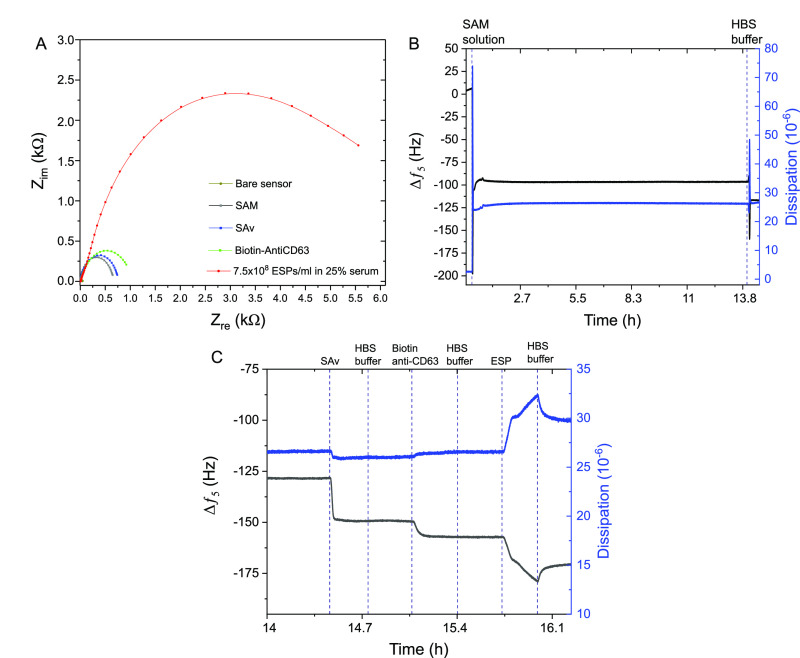
Example of tandem EIS and QCM-D information acquisition. (A) EIS response across entire sensing process (fabrication to exosome-sized particles (ESP) detection) captured simultaneously alongside, (B) QCM-D response profile on the same WE for SAM formation (from ethanolic solution) and the addition of (C) SAv, biotin-anti CD63 and 7.5 × 10^8^ ESPs mL^–1^ in HBS buffer. Vertical dashed lines represent the start of respective sample uptake by the peristaltic pump.

