# Supplementary material for: Dual-Mode and Label-Free Detection of Exosomes from Plasma Using an Electrochemical Quartz Crystal Microbalance with Dissipation Monitoring
Source: Anal Chem. 2022 Jan 24;94(5):2465–75. doi: 10.1021/acs.analchem.1c04282 (PMC9096790; doi:10.1021/acs.analchem.1c04282)
Supplement: Supplementary file 1 — ac1c04282_si_001.pdf [file ac1c04282_si_001.pdf]

-SUPPORTING INFORMATION-

Dual-Modal and Label-Free Detection of Plasma Exosomes using an Electrochemical Quartz Crystal Microbalance with Dissipation Monitoring

Jugal Suthar,[†,‡] Beatriz Prieto-Simon,[¶,§] Gareth R. Williams,[†] and Stefan Guldin[*,‡]

†*UCL School of Pharmacy, University College London, 29-39 Brunswick Square, Bloomsbury, London, WC1N 1AX, United Kingdom*

‡*Department of Chemical Engineering, University College London, Torrington Place, London, WC1E 7JE, United Kingdom*

¶*Department of Electronic Engineering, Universitat Rovira i Virgili, 43007, Tarragona, Spain*

§*ICREA, Pg. Lluis Companys 23, 08010, Barcelona, Spain*

E-mail: s.guldin@ucl.ac.uk

Table S1: Supplementary information contents table

Supporting Item	Page number
Western Blot methodology	S2
Figure S1: Characterisation of SEC fraction isolates.	S3
Figure S2: Evaluation of optimal electrochemical cell model for EIS fitting	S4
Table S2: Goodness of fit (P-value) model comparison (alpha level: 1%)	S5
Figure S3: EQCM-D performance against varying concentrations of ESPs spiked in HBS buffer.	S5
Figure S4: EQCM-D response against ESPs in HBS buffer and 25% serum using a control sensor.	S6
Figure S5: Measurement mode comparison of SNR across titrated ESP concentrations.	S6
Figure S6: EQCM-D comparison between control and target sensors at LOD concentrations.	S7

Western blot methodology

40 µl of deionized water was added to dithiothreitol (DTT) to make a 400 mM solution. 10 µl of SEC fraction 4 was lysed in 20 µl RIPA buffer as described above. The lysed solution was then diluted to a working concentration between 0.2-1.0 µg per µl with 0.1X sample buffer, before 4 µl was mixed with 1 µl of loading buffer (flourescent 5x master mix). The loading buffer was prepared by adding 20 µl of 10x sample buffer, and 20 µl of 400 mM DTT solution. Since the detection of tetraspanin proteins requires non-reducing/native conditions, an additional 20 µl of 0.1X sample buffer was added in place of the DTT solution for these samples. Thus, Alix, CD9 and CD63 proteins were detected in separate capillaries. The samples and biotinylated ladder were then denatured on a 95 °C heat block for 5 minutes, before being briefly centrifuged and loaded onto the detection module assay plate. Anti-Alix, anti-CD9 and anti-CD63 primary antibodies were used at 1:20, 1:10 and 1:10 dilutions respectively and loaded onto the primary antibody. Corresponding mouse-reactive secondary antibodies were then added, followed by the chemiluminescent substrate, comprising 200 µl luminol-s and 200 µl peroxide. The fully loaded plate was then centrifuged at 2000 RPM for 5 minutes before being inserted into the WES system, in conjunction with a 13-capillary cartridge. Detection and quantification was conducted via a CCD camera and the Compass software, version 3.1.7 (Protein Simple, USA). Anti-Calnexin (W17077C, Biolegend) was used as a negative control to ensure that no cellular protein was present in the chosen SEC fraction.

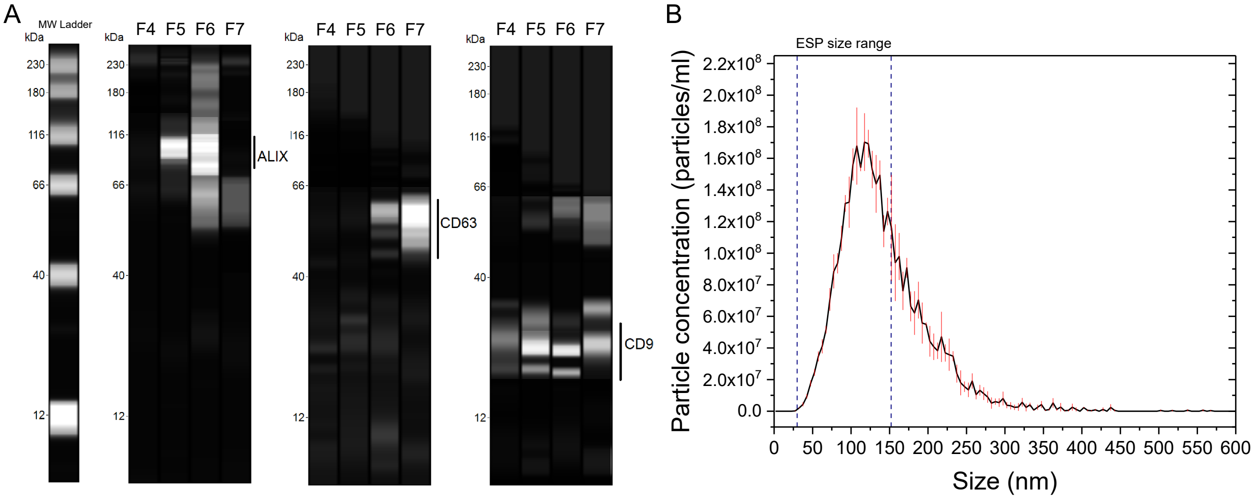

Figure S1: **Characterisation of SEC fraction isolates. (A)** Western blot analysis of SEC fractions 4, 5, 6 and 7 from human plasma, probing for exosome enriched proteins Alix, CD63 and CD9. **(B)** Particle size distribution profile of SEC fraction 6 as determined by NTA.

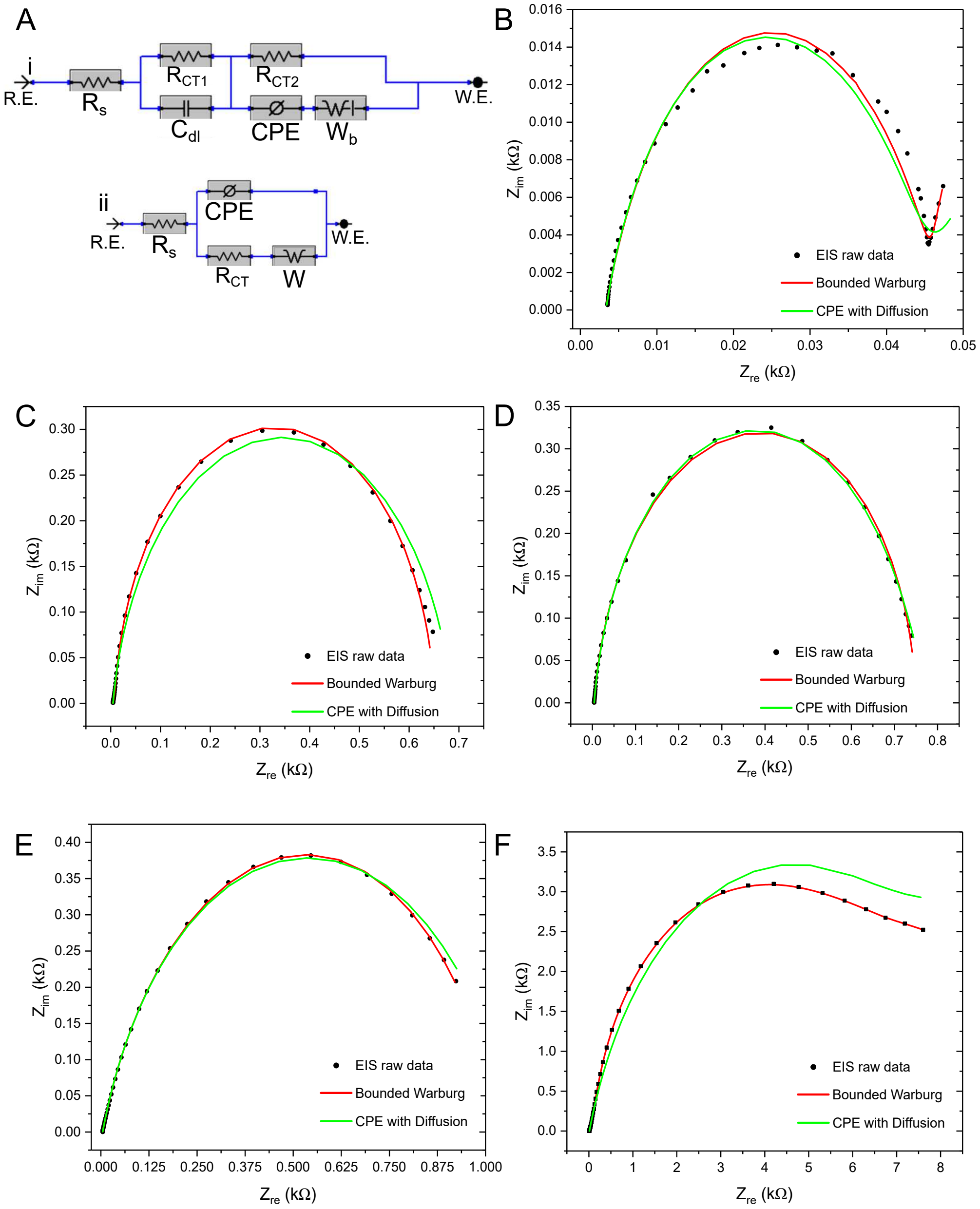

Figure S2: **Evaluation of optimal electrochemical cell model for EIS fitting. (A)** Circuit diagrams representing two cell models: *(i)* Bounded Warburg model and *(ii)* CPE with diffusion model. Parameter definitions: Rs, resistance of the solution; Rct, charge-transfer resistance; W, Warburg element corresponding to the diffusion effect; CPE is an "effective" parameter to correct the non-ideal behaviour of the double layer; C (Cdl), dielectric layer after exosome binding; and R (Rct2), resistive effect of the exosomes. Nyquist plots showing fittings of both models to EIS response after addition of each sensing layer; **(B)** bare sensor, **(C)** SAM, **(D)** SAv, **(E)** anti-CD63 and **(F)** $1x10^9$ ESPs/mL.

Table S2: Goodness of fit (P-value) model comparison (alpha level: 1%)

Layer	CPE with diffusion	Bounded Warburg
Bare sensor	9.5×10^{-4}	7.97×10^{-4}
SAM	5.6×10^{-3}	1.6×10^{-3}
Antibody	8.5×10^{-4}	8.2×10^{-4}
SAv	1.1×10^{-3}	3.5×10^{-4}
Exosomes (1×10^9 ESPs/ml)	5.6×10^{-3}	9.8×10^{-4}

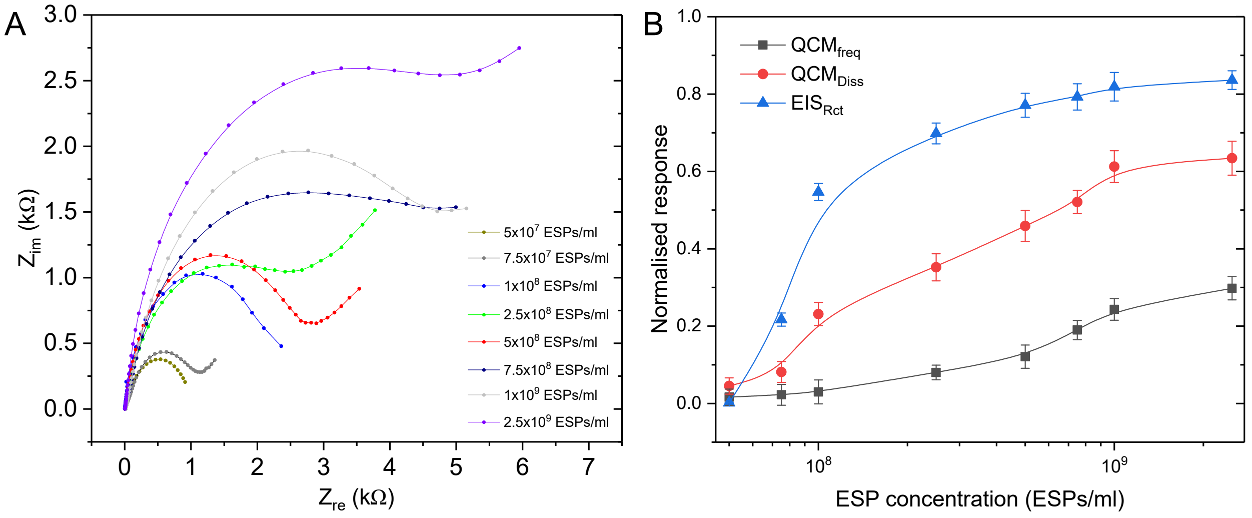

Figure S3: **EQCM-D performance against varying concentrations of ESPs spiked in HBS buffer on a target sensor.** **(A)** Nyquist plots representing EIS response. **(B)** Normalised response changes across EIS, frequency and dissipation.

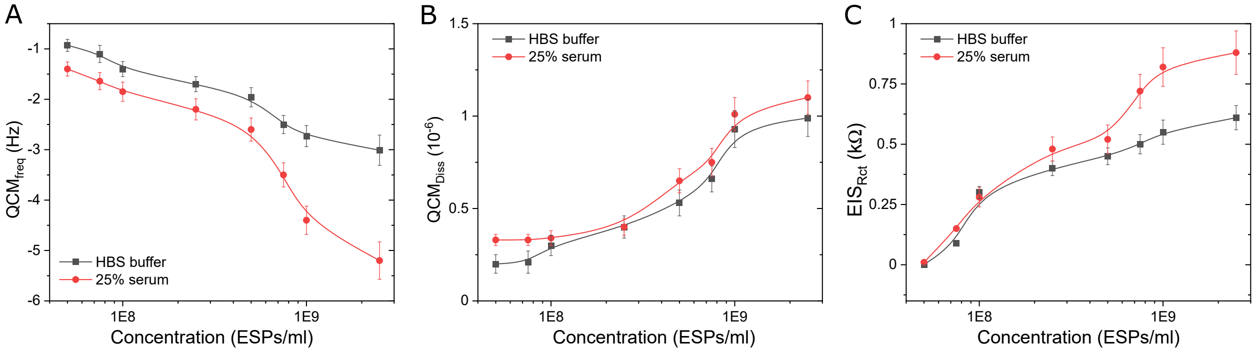

Figure S4: **EQCM-D response against varying concentrations of ESPs spiked in either HBS buffer and 25% serum using an isotype control sensor. (A)** Frequency response, **(B)** dissipation response and **(C)** EIS response on control sensor surfaces. Standard deviation determined from three independent experiments.

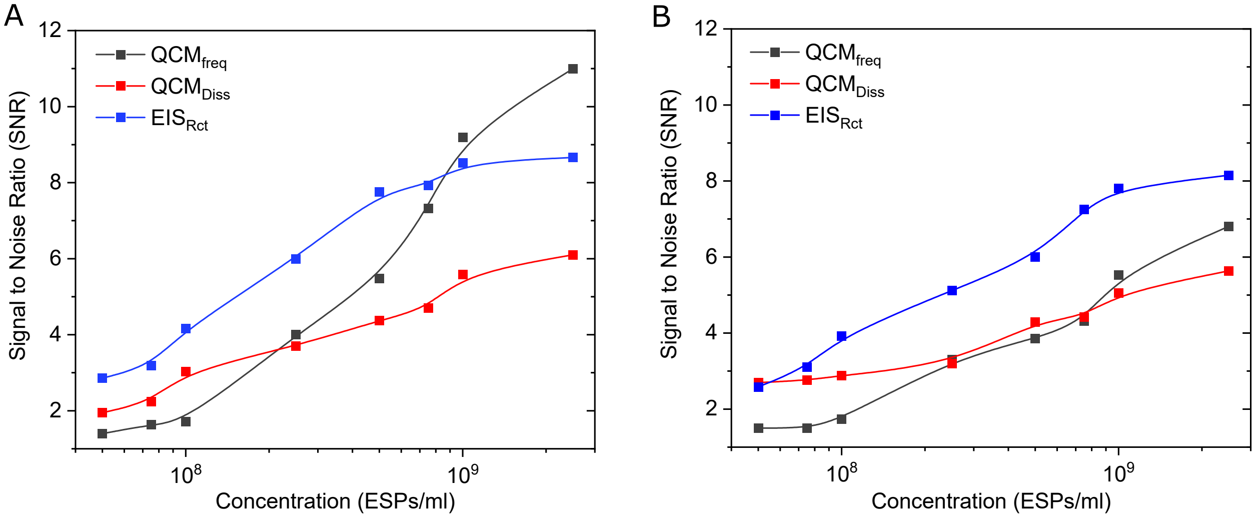

Figure S5: **Measurement mode comparison of signal-to-noise ratio (SNR) across titrated ESP concentrations.** Determined SNR for ESP samples in **(A)** HBS buffer and **(B)** 25% serum.

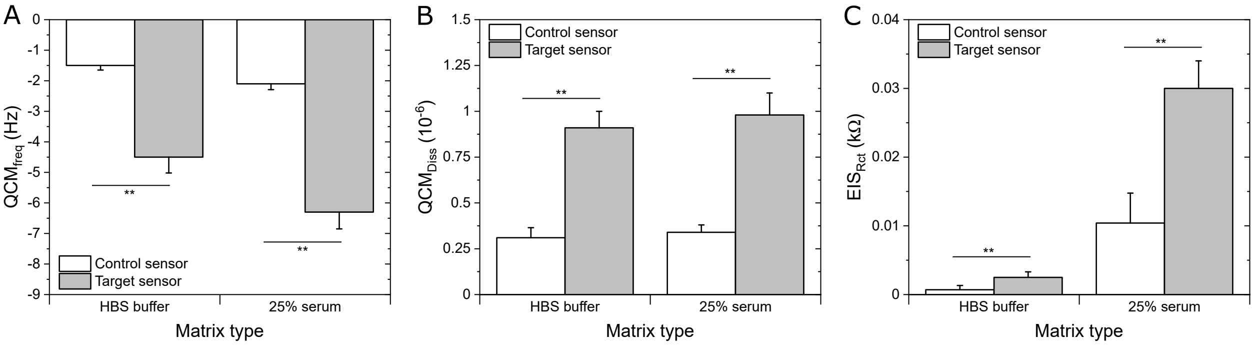

Figure S6: **EQCM-D response comparison between control and target functionalised sensors at each measurement mode's respective LOD concentration in HBS and 25% serum.** **(A)** Frequency response comparison, **(B)** dissipation response comparison and **(C)** EIS response comparison at LOD concentrations in both 25% serum and HBS buffer. Standard deviation determined from three independent experiments. Student t-test (two-tailed) compared responses from the control and target sensor (**$p < 0.01$).
